# Use and discontinuation of intrauterine contraceptive device in the Greater Accra Region of Ghana

**DOI:** 10.1186/s40834-018-0061-1

**Published:** 2018-06-06

**Authors:** Fred Yao Gbagbo, Esinam Afi Kayi

**Affiliations:** 1 University of Education Winneba, Box 25, Winneba, Ghana; 2 Regional Institute for Population Studies, University of Ghana, Box LG 96, Accra, Ghana

**Keywords:** Use, Discontinuation, Intrauterine contraceptive device, Greater Accra Region, Ghana

## Abstract

**Background:**

The intrauterine contraceptive device is one of the modern long-acting and reversible contraception that is very safe and effective. Yet, less than 2 % of women are using intrauterine devices in Ghana. This study therefore explored the experiences and barriers to intrauterine contraceptive device use and discontinuation in Greater Accra Region, Ghana.

**Methods:**

Intrauterine contraceptive device users and providers were purposively selected from eight private family planning clinics in the Greater Accra Region. Semi-structured interview guides were used for in-depth interviews during data collection. The interviews were audio-taped to ascertain accurate accounts of the interviews and recordings replayed for analytical responses. Field assistants transcribed the interviews conducted themselves and read through the transcripts produced twice to increase familiarity with the dataset. A list of code labels was created and a series of categories for the main themes that emerged from the transcripts were developed. The transcribed data was organized, coded and manually thematically analysed in word. Study results were presented in tables and quotes from respondents.

**Results:**

Results showed that key motivations for intrauterine contraceptive device use include effectiveness, benefits, and efficacy of the device, fertility regulation, peace of mind, contraceptive method switching, health provider effects, desire for long-acting contraceptive method, and partner characteristics. Intrauterine contraceptive device discontinuation was due to bleeding irregularities, vaginal infections, desire to increase fertility, physical features of the intrauterine device, and partner disapproval of use. Other reasons in both cases pertained to non-hormonal aspects of the intrauterine device, partner characteristics, and provider encouragement and influence.

**Conclusions:**

Several factors influence the use and discontinuation of intrauterine device in Ghana. Comprehensive contraceptive counselling on the intrauterine device is essential in promoting uptake and knowledge of the intrauterine device at the health facility level. Various targeted messages are also needed to dispel misconceptions at the community level.

## Background

Globally, the intrauterine contraceptive device (IUD) has been recognised as one of the modern long-term reversible contraceptive methods suitable for women of all reproductive ages [1]. It represents the most cost effective reversible method for preventing unwanted pregnancies [2]. Scientifically proven for its safety, efficacy and cost-effectiveness [3], the copper T380A and Levonorgestrel releasing IUD is known to be more effective and longer lasting in preventing pregnancy than tubal sterilization, pills, patches and contraceptive rings [4, 5].

A number of studies show that several factors account for the poor IUD use among women [6–8]. These factors relate to healthcare provider characteristics, health system and individual or user factors [9, 10]. While these may be enough, sociocultural norms, beliefs and practices may serve to regulate IUD use even among high risk women or sub-groups particularly in Africa where there are entrenched sociocultural norms, beliefs and practices on childbirth [10].

Health provider characteristics that contribute to low IUD utilisation include misperception that IUD is associated with an ongoing risk of pelvic inflammatory disease (PID) and resultant infertility which particularly hinder IUD use among nulliparous women, especially if they are single or have several sexual partners [11]. In addition, few health care providers are shown to be hesitant to insert an IUD into nulliparous women because of perceived technical challenges [12].

Individual factors which prevent IUD uptake are often accentuated by misconceptions, beliefs and myths. Whilst women report conceptual concerns and fears about having a foreign body placed inside their womb, a lack of counselling and adequate information about IUDs from healthcare providers to enable them make informed decisions [13], fear of painful insertion [14, 15] and the perception of IUDs as abortifacients, risks of PID and ectopic pregnancy [16] deterred IUD use.

Although IUDs are considered as one of the most popular long acting and reversible contraceptive methods worldwide [17], they are used by less than 2% of Ghanaian women in their reproductive age [18]. Ghana’s Demographic and Health Surveys (GDHS) have all shown very low IUD usage among both married and unmarried women [19]. Whilst reasons for low IUD use are poorly understood, the DHS does not provide an in-depth understanding of the factors or reasons associated with low use because the methodology is purely quantitative. This study therefore aimed to explore women’s knowledge and perception about IUD, reasons for IUD use, barriers facing IUD uptake and provider characteristics influencing IUD using a qualitative method to provide in-depth information that will complement the GDHS data on IUD to inform policy and programme interventions aimed at increasing IUD uptake in Ghana.

## Methods

### Study design

The study employed a retrospective and cross-sectional qualitative design with an in-depth interview to obtain, analyze, interpret and report data. This was adapted from a previous study that used a cross-sectional designed [20]. The purpose of using this qualitative design in this study was to explore a range of opinions and its different interpretations with the aim of maximizing the opportunity to understand the different positions taken by different members of the social milieu. This design therefore enabled an in-depth exploration of women’s experiences with the IUDs as represented differently from their own perspective.

### Study population

Women in their reproductive ages (15–49 years) who had ever received family planning (FP) services, and particularly, IUD from the study facilities were selected to participate in the study. Women who were eligible to partake in the study were: Non-IUD users, current IUD users and women who had ever used IUD. The justification for choosing these women was because their experiences for not using, using or discontinuing the IUD use helped to answer the research questions. They were also most appropriate individuals to provide rich detailed information on IUD use. Trained and practicing IUD service providers (midwives and medical doctors) from the family planning units of public, private and NGO health facilities were also purposively interviewed to obtain divergent opinions on their experiences with FP choices including IUDs.

### Study setting

The study was conducted in six renowned health facilities (2 public, 2 private and 2 NGO) that provide family planning services within the Greater Accra Region. The selection of these facilities was purposive. This was based on complete family planning data availability from the regional health directorate and the facilities client information management systems from 2008 to 2015. These facilities so far have not examined factors affecting uptake, satisfaction and acceptability of reversible family planning methods, hence creating a gap between client demand and provider supply which justifies the need to conduct the study in this setting to inform FP decisions.

### Ethical considerations

Written informed consent was obtained from all interviewees for their participation and for the audio-recording of the interviews. Interviews were conducted in private places such as consulting rooms, respondents’ homes and offices as recommended by respondents to protect the confidentiality of responses and enhance the comfort of respondents. This study received ethical approval from the Ghana Health Service (GHS) Ethical Review Committee (ERC). Permission was also sought from the various health facilities and respondents used in this study.

### Data collection procedure

Data collection began using a sample frame. A list of women who had visited the identified health facilities for IUD insertion or removal during the past six months were obtained from the client information system after several unsuccessful attempts to directly recruit the required number of participants at the study sites. Considering the general low patronage of IUDs at the study site during the period of data collection, it was very challenging obtaining the required number of respondents for a meaningful analysis hence the need to fall on client records for retrospective interview of current and previous IUD users from the indicated facilities. Clients using other modern contraceptive methods were also interviewed to explore why they are not using IUDs but other methods. Family planning providers offering modern family planning methods, including IUD insertions and removal in the selected facilities were also interviewed to examine their experiences about clients concerns with providing IUD services.

Participants were recruited either by face-face or through phone calls after they were informed about the nature, benefits, risks, and purpose of the study and consented to participate. Information on voluntary participation, rights to withdraw and consent was made known to study respondents. Half of the in-depth interviews (IDIs) were conducted at health facilities, whilst the remaining were held at respondent’s home, and at places deemed convenient and comfortable for respondents.

Data were collected using modified research instruments to build upon previous literature. In line with the study objectives and issues identified in the literature reviewed, four (4) different semi-structured interview guides were developed to address the research questions. The interview guides contained questions on participants’ socio-demographic data (such as age, educational level, current FP use, previous FP method use, and parity); FP knowledge, and knowledge about IUD, and factors/reasons for IUD use. Questions for IUD users’ differed slightly from questions asked of women who had ever used the IUD. For instance, whilst IUD users were questioned on their perception about the IUD when they initially began using it, women who had removed the IUD were asked to report on reasons for their removal of the IUD, side-effects, and future intentions to use the IUD. Thus, although questions differed slightly on some respects, a few similar questions were asked to answer the research objectives. Family planning providers were interviewed on the nature of their relationship with clients, clients’ knowledge on the IUD and clients reasons for discontinuation of the IUD.

Three female research assistants were trained by the lead investigator to administer the interview guides. Although they were encouraged to be flexible during data collection, strict adherence to the study guide was discouraged if new topics in relation to originally stated questions emerged during the discussions. This flexibility was to encourage capturing of emerging issues which are different from the order given in the interview guides [21]. The field research assistants were also oriented on key ethical issues regarding research ethics involving humans as research subjects and were encouraged to be nonjudgmental in their responses to the experiences of the study participants. Face to face in-depth interviews were conducted in the local language ‘Twi’ or in English. Nine (9) study participants were interviewed in ‘Twi’ and the rest were in English. The In-depth Interviews lasted for an average of 40 min.

The interviews were audio-taped with permission from participants to ascertain accurate accounts of the interviews. The recordings were then replayed for analytical responses. Interviews were transcribed immediately thereafter while ‘Twi’ interviews were translated to English and later transcribed. Field assistants transcribed the interviews they conducted themselves. As the transcripts were produced, they were read through to increase familiarity with the data. Data was manually analysed by the researchers using the thematic analysis approach. To do this, a list of code labels was created and a series of categories for the main themes that emerged from the transcripts were developed. The transcribed data was then organized, coded and manually thematically analysed in word. Results of the study were then presented using descriptive statistics and quotes from respondents.

## Results

Table 1 presents the socio-demographic characteristics of respondents excluding FP providers. For IUD users, six (6) were aged between 40 and 49 years, and the remaining were between 25 and 29 years. Two (2) out of the ten participants had no formal education. Only one woman was unmarried but she had two (2) children. Majority (8) had been using the IUD for more than six (6) months; only two (2) women had used it for two months and five months respectively. Twelve (12) women had ever used the IUD for duration of two weeks to thirteen (13) years. Their current FP methods comprised of both modern and traditional FP methods such as, implants, injectable, condom, withdrawal and the calendar method. Two (2) were not using a method; one was pregnant, and three were trying to get pregnant. Amongst these women, one didn’t complete primary education, two had completed senior high school, and the rest had tertiary education. Two (2) participants worked in the public service, six (6) worked in private organizations, and four (4) were self-employed.

**Table 1:** Socio-demographic characteristics of study respondents

Characteristic	IUD users	Ever IUD users	Non-IUD users	Total
	*n* = 10	*n* = 12	*n* = 7	*N* = 29
Age				
20–25	2		2	4
26–30	2	3	3	8
31–35		3	2	5
36–40	1	3		4
41–45	3	1		4
46–50	2	2		4
Educational status				
No education	2			2
Primary	1	1	3	5
Secondary	3	2	4	9
Tertiary	4	9		13
Marital status				
Unmarried	1	2	1	4
Married	9	9	6	24
Divorced/separated				
Widowed		1		1
Occupation				
Unemployed			1	1
Self-employed	7	4	4	15
Public worker	1	3		4
Private formal worker	2	5	2	9
Parity				
1	1			1
2	2	3	1	6
3	3	4	3	10
4	2	3		5
5	2			2
6+				
Current FP method				
None		2	2	4
Trying to get pregnant/ pregnant		4	2	6
IUD	10			10
Implant		1	2	3
Injectables		1	1	2
Male Condom		1		1
Female condom				
Withdrawal		1		1
Calendar method		2		2
Tubal ligation				
Number of years using current FP				
Less than 6 months	5	3	3	11
6 months −1 year	3	3		6
2 years- 5 years	2			2
6 years–10 years				
More than 10 years				
Previous FP method				
None	3		1	4
IUD	2	11		13
IUS		1		1
Implant	1		2	3
Injectables	1		1	2
Pills	2		1	3
Male Condom				
Female condom				
Male condom				
Calendar method	1			1
Tubal ligation				
Number of years used previous FP method				
Less than 6 months	1	3		4
6 months −1 year	2	5	1	8
2 years- 5 years		3		3
6 years–10 years			2	2
More than 10 years	1			1
Total				28

From the total IDIs conducted, seven (7) respondents had never used IUD as an FP method. Their ages ranged between 25 years to 32 years. Only one (1) was not married. Two (2) were currently using the implants; one (1) was using injectables; two (2) were trying to get pregnant, and the remaining two (2) were not using any FP method. The highest educational status attained by respondents in this group was secondary, (four respondents) and three (3) had completed junior high school. Almost all women had at least two (2) children, except two women who were trying to get pregnant.

Seven (7) IUD providers participated in the IDIs. Three out of the seven from NGO facilities whilst the remaining four (4) were from private (2) and public (2) facilities. The professional qualifications of FP providers ranged from Health assistant, Midwife, Nursing officer, Obstetrician and gynaecologist. There was a sole male FP provider in the study who worked as an Obstetrician. All providers have been providing FP services for more than 5 years except the health assistant who was not professionally trained and specialized to offer IUD insertions for women. Her responses are therefore not included in the analysis.

A total of thirty-six (36) IDIs were conducted. Out of this number, ten (10) women were currently using IUD, twelve (12) had ever used the IUD, and seven (7) had never used the IUD. The remaining seven (7) were family planning (FP) providers. Majority (12) of interviewees were selected from NGO facilities. Of the FP providers interviewed, only one was not providing IUD services. Her responses are therefore not included in the analysis. The number of IDIs conducted at each study setting is presented in Table 2.

**Table 2:** Number of IDIs conducted at each study setting

Study group	Facility 1	Facility 2	Facility 3	Facility 4	Facility 5	Facility 6	Total
IUD users	3	2	2	1	1	1	10
Ever IUD users	4	1	1	2	1	3	12
Non-IUD users	2	–	1	1	2	1	7
FP providers	3	1	1	1	–	1	7
Total	12	4	5	5	4	6	36

### Knowledge of family planning

Findings showed that all women had good knowledge about FP methods. Each study participant knew of at least, three FP methods and how it works. The commonly mentioned FP methods were injectables, implants, and pills. However, half of the women could not provide the name of the implants by themselves, but were able to describe where and how it was inserted. Very few (three) respondents mentioned sterilization (vasectomy and tubal ligation), diaphragm. One woman mentioned IUS. Furthermore, the interviews showed that IUD users and ever IUD users had adequate knowledge of the IUD compared to women who were not using IUD. Study respondents’ level of knowledge about the mechanism of action (i.e. how the IUD works) of the IUD, description of its physical features, and who can use it was comparatively the same among current and previous IUD users.

### Findings from current IUD users

From the IDIs, only ten (10) women were currently using the IUD. Out of the ten women, five (5) voluntarily decided to use the IUD whilst the remaining were given the IUD as a post-abortion contraception method. Three women who voluntarily preferred to use the IUD stated it was their personal decision; they were not encouraged or motivated by family, friends or staff, whilst the other two admitted being motivated by their friends, and health providers. A respondent categorically reported that her desire to use the IUD was out of a strong personal volition irrespective of her partner’s consent. A respondent said:

*“Nobody encouraged me to use the IUD. My husband, mum and siblings even did not want me to do the FP since they were all afraid of the side effects. So it was my own decision. I just got up one day and decided to go and do it because my children have very short intervals in relation to age. So even my husband doesn’t know I’ve done FP unless I tell them” (29 year old married woman currently on copper-T IUD for 1 year).*

Although the walked-in clients reported to have voluntarily consented for IUD based on the counselling they received, clients who had the IUD following post-abortion care reported to have been coerced/forced/encouraged/influenced to have an IUD as post abortion contraception to avoid repeat unwanted pregnancy and abortion. Two respondents mentioned that they were respectively encouraged and influenced to use the IUD by the provider after having undergone an induced abortion. A respondent stated that:

*“I refused an immediate family planning uptake despite the in-depth counselling given me after the previous abortion. So when I went and got pregnant and came again for another abortion, she said by force she will do one for me so I agreed for her to do it for me although it was against my wish” (40 year old married woman on Copper-T, IUD for1 year).*

Half of the participants reported no side effects after switching from injectables to having the IUD inserted. Again, another half felt slight abdominal pains soon after insertion of the IUD which was relieved after ingesting pain killers given by the provider. One respondent stated:

*“I went back to the IUD because for me it was very successful. I never had any problems with the IUD compared to when I was using the injectables. I didn’t have any problems with my period, I didn’t have any weight issues and headaches compared to the injectables that I previously used” (43 year old unmarried woman using Copper –T 380A for 2years)*

Another respondent indicated that:

*“my experience so far is that my menstrual flow has reduced from 5 days to 4 days since using the IUD for six months now after I delivered my last baby. I decided to ask a doctor friend and he said nothing is wrong with me. I also realized, comparing this with the injectables that I used to be on, the severe heart beat and headache I used to have disappeared. Initially, I was a bit tensed, that maybe my husband will find out. So one day, I asked him about it whether he can feel anything. And he said no. So he asked is it like the other ones? I said no, your menstrual cycle is it normal? I said normal, regular 28days cycle. I said I’m ok. I don’t know; I’m not worried. I feel normal. I feel ok. So I’ve been encouraging some of my colleagues to do the IUD” (29year old married woman on Copper T 380 A for 2years)*

In another instance, one participant indicated having prolonged menstrual flow and offensive vaginal discharges. According to her, the duration of menstruation is slightly longer and heavier compared to when she had not inserted the IUD. She narrated her experiences as follows:

*“You are just there and then you get a discharge. It’s not too comfortable, but then, I initially tried to cope until the discharge became offensive which compelled me to seek medical help that advised me to remove it after two months of unsuccessful treatment.*

*I realize that, even though it is just about two months since removing the IUD, It’s a little better. Now the discharges come at the time of ovulating. Although I realize that there are more discharges it’s no longer offensive. I learnt it’s going to go back to normal after a while” (42 year old married woman wo used Copper-T 380A for 2months but currently on combined oral contraceptive pills).*

Another respondent asserted that her menstrual flow is now irregular compared to when she hadn’t inserted the IUD. Also, she initially experienced some brief periods of vaginal discharge, which did not smell but currently doesn’t have any discharge. She indicated that:

“……I later realized that some ‘water* (referring to discharge) comes from my vagina. But they told me that when we see some water and we don’t like it we should come back. But I didn’t go back and the water stopped. Since then I have not experienced any major problem just that my menses is not flowing like it should be.” (46 year old married woman on Copper-T 30A for 1year).

Two respondents reported that this was their second time of using the IUD. Both of them reported no side effects on both occasions. According to one participant, she removed the IUD to test her fertility and continued usage after childbirth as encouraged by her doctor’s explanation and counselling. She narrated her story as follows:

*“….So he said that IUD will be the best solution so I went into that. I used that for about 6 years, I met this guy we talked about marriage and he was like the thing that people are telling him is that if you take it off you won’t get pregnant again. He insisted that before we get married I should get pregnant for him first. So I took it off and became pregnant the following month and got married but aborted the pregnancy on health grounds after which I had another replaced for 3years. I took it out again and I got pregnant again (laugh) after I gave birth, I read about LNG-IUS and I went to the hospital requesting for it because of its advantages. But I was told it is not available in Ghana yet. I was like okay am not going to have any babies any time soon so let me go back to my Copper IUD. So I had it again for almost 10years now.” (43year old unmarried woman on Copper-T 380A for 10years).*

### Factors that influence IUD uptake

Table 3 presents an illustrative summary of factors influencing IUD uptake in order of most frequently mentioned factors. Respondents gave varied reasons for current use of IUD compared to other methods. The main reasons reported to have determined IUD uptake pertained to protection against unwanted pregnancy, to reduce births or space childbearing.

**Table 3:** Illustrative summary of factors influencing IUD use among current IUD users

No.	Factors influencing IUD uptake	Frequency	Percentage (%)
1	Prevention of unwanted pregnancy	20	21
2	Reduce births	17	18
3	Space births	15	16
4	Prevent abortion	11	11
5	Contraceptive failure	10	10
6	Avoid forgetfulness in taking pill	6	9
7	Substitute to other FP methods due to unpleasant experiences	5	5
8	Desire for a long lasting permanent method	4	4
9	Concentrate on work, or business	3	3
10	Non-hormonal method	2	2
11	Concentrate on academics	1	1
Total		94	100

Other factors that influenced IUD uptake were centred on the characteristics and benefits of the IUD over implants, pills and injectables. Half of respondents’ decision for using the IUD was premised on past contraceptive failure and a desire for a long lasting family planning method. In a narrative account, a study participant’s decision to adopt the IUD was due to ineffectiveness of traditional natural FP method; to avoid unpleasant previous experiences with other FP methods; fear of partner’s reaction to successive pregnancy, and desire for a different FP method other than implants, injectables or pills in order to avoid another repeated abortion. She admits being encouraged by the provider to use IUD during post-abortion counselling. The need to avoid unpleasant side effects with the pills, injectables as well as forgetting to take the pills and ease of use influenced IUD uptake. Two women reported:

*“I had issues with the tablet because I kept forgetting yes and your menstrual cycle change with it and all that. That is why I went for the IUD” (39year old unmarried woman on Copper-T IUD for, 2years)*

Another respondent indicated that:

*“I had wanted to do the 5years type of IUD but it wasn’t available. I was told the 5years type of IUD, the implants and 3 months injectables are almost the same. Since I had done the 3 months and felt dizzy, I decided to do the 10 years IUD because that one isn’t medicine but just something to close the vagina” (29year old married woman, using copper-T 380A for 2years)*

Two respondents were convinced about the efficacy of the IUD influenced their choice of the IUD as a method to prevent pregnancy. One said: *“It was effective for me previously and it’s still the best for me” (23 year old married woman on Copper-T 380A for 1 year).*

Another also said: *“I was more convinced about the efficacy of it the choice” (43 year old unmarried woman, on Copper-T 380A for 2 years).*

In furtherance to the reasons for IUD use, two women also decided to use IUD because of its non-hormonal nature which does not affect their hormonal system. In their view, the IUD was the best method. One noted in the following:

*“IUD is the best method because, I think it does not have any hormonal thing when you do it unlike the one that you inject or has something to do with your blood. Those ones I hear people have complications, hormonal disturbances, I think IUD is best because it just blocks your womb and no sperm enters to have an egg fertilized”* (*23year old married woman, on Copper-380A for 2years.*

Partner characteristics also determined preference for IUD use in a few cases. For one woman, the decision to use the IUD was fear of partner’s reaction to successive pregnancy, and to avoid problems with partner. Narrating her story she said:

*“I became pregnant for the fourth time and I fear my husband will complain and refuse my education. My husband suggested an abortion and IUD. He encouraged me this is the best after the abortion which I did” (29year old married woman, on Copper-T 380A for 3 year).*

Another respondents indicated that:

*“I already have four kids and my husband is giving me problems to have more. if I tell him about family planning he would not allow me to do so I came to do this on my own secretly” (44year old married woman on Copper-T 380A for 2years).*

The insistence and encouragement of some health providers led to IUD uptake for some women. A respondent categorically admitted being encouraged by providers to use IUD after post-abortion counselling. They claimed providers did not directly influence their choice, but rather advised on the need for adopting an effective method to prevent repeated abortions. A respondent asserted in the following:

*“----So when I went and got pregnant and came again and she said by force she will do one for me so I agreed for her to do it for me”….(40year old married woman, 3 children on Copper T 380A for 3years).*

Another respondent indicated that:

*“ ---midwife insisted on me taking an IUD after doing the abortion else she will not help me again. Because of the respect I had for her, I had the IUD soon after the abortion for free” (28year old widow, 2 children on Copper T 380A for 1year).*

### Perceptions about IUD

Study participants’ indicated to have had mixed reactions when first introduced to the IUD. Majority (8) of the respondents said they were scared when they first heard about the IUD. The insertion procedure created fear amongst them hence did not opt for an IUD as FP method. Two respondents explained:

*“I have heard about FP before. They say some FP is put in the arms, some too were injections and some too were inserted in the womb. But I didn’t know how it looks like. So when I came and it was explained to me I was scared so I didn’t do it but opted for the injection” (40 year old married woman on injectables).*

The other respondent indicated:

*“Yes, I was very afraid; because I had not done some before. I’m a person full of fear. I taught of something different. But the provider took time to counsel me and even used models for demonstrations on how it is done. Despite my fears, I tried it. I taught it will be painful for months but nothing of that sort happened” (25 year old married woman, on Copper-T 380A for 3years).*

Women’s perception of the IUD was also associated with myths and negative reactions from friends. They raised concerns about some myths that they had heard about the IUD getting stuck in the womb, and potential health risks in later years. For respondents who associated myths and misconceptions about the IUD, they affirmed that their perceptions had now changed. A respondent reported that her lack of knowledge about how the IUD works, and how it looked like made her afraid. The fact that “something” was going to be inserted in the womb created fear, because her understanding of the procedure is similar to undergoing a D & C. However, her perception changed after explanations from the provider. She explained:

*“Oh in the beginning when they were about to fix it and it scared me a bit because I didn’t know how it will work or how it is. Ehee, but they made us understand that it wasn’t anything scary. Something that they said they will put in your womb, you will be a bit scared” (46year old married woman on Copper –T 380A for 5months)*

Other respondents who did not discuss their FP choices with their partners, feared that their partners might find out about having the IUD since the strings were likely to be visible. One respondents indicated that:

*“Initially, I was a bit tensed, that maybe my husband will find out. So one day, I asked him about it whether he can feel anything in me during sex. And he said no. I then became comfortable keeping it all these years without him knowing” (29year old married woman on copper-T 380A for 5 years).*

Some respondents were just curious and desirous to try a longer lasting FP method when initially informed about the IUD by a health provider. Other respondents were further motivated to use the IUD with positive assurances and encouragement from friends. Further, another woman also expressed indifference when initially informed about it. She said: ‘*I just wanted to protect myself, I didn’t have any attitude towards it’ (29 year old married woman, on Copper-T 380A for 2 months).*

### Perception of health risks associated with using the IUD

In Ghana, knowledge about Family Planning methods is generally acquired though formal and informal public education. Whereas the educated population read about family planning methods when the need arises, the less educated obtain family planning information in health facilities, friends and significant others including television, radio and social media adverts. When respondents were asked whether using the IUD was associated with health risks, seven (7) reported no health risks associated with using the IUD, while one (1) respondent couldn’t tell whether or not the IUD posed a health risk. She indicated:

*“I was not scared of any risk, because I didn’t think there will be any. But there was one thing that was at the back of my mind, that if I am not comfortable with it, I will take it out. Do you know, if I am not comfortable I didn’t think that they could do something, you know negative, but all I was thinking is that, if I am not comfortable, I will take it out” (42year old married woman, on Copper–T 380A for 5 years)*

A respondent admitted that no health risks were possible except risk of getting infected through usage of unsterilized equipment. However, one woman feared being sterilized after using the IUD. Another participant had no knowledge about whether IUD usage could harm her in future. She explained:

*‘If there is a development that it affects something because the copper something scared me, because I didn’t understand why you should have metal in you but I googled and read about it and I think its ok, it doesn’t have any health risks’ (23year old unmarried woman on Copper-T 380A for 3 months).*

Although majority (7) of the women interviewed felt safe with using the IUD, a few (five) disliked the uncomfortable feeling of the strings hanging in vagina during menses, bathing and somehow wished the string will be ‘cut off’. Three of them had this to say:

*“Yeah the little thing that I don’t like about it is that, when you are having your menses the bleeding brings the strings out. They put the “T” thing in the womb, and then they leave this (referring to the string) to hang out into the vagina. So as you, you know, if, mensuration stops it sort of goes in to…“Aha”, When you are taking shower and your hand just goes there, you can feel it. I would have wished they will even cut my string out” (42year old married woman, on Copper –T 380A for 5 years).*

Another respondent explained:

*“oh the first time I fixed it, the thread was lying around my vagina so I was afraid so I was pushing it in, then I later went there to complain about it. They removed it for me and then fixed another IUD for me but still there’s some thread lying around my vagina. Anytime I wash that area, I feel it but now I don’t care” (23 year old married woman, on LNG-IUS for 1 year).*

Another respondent was of the view that:

*“the previous IUD I inserted, I don’t know what happened maybe I had a heavy flow in a particular month and it came out and you know it has a string attached to it, so I think it was choking me around my cervix and when I was sitting, it was so down, I was feeling a little pain that’s why I went to change it; but apart from that, I’m ok with the current one as its very comfortable” (29year old married woman, on Copper –T 380A for 2 years).*

### Findings from women who have ever used the IUD as post abortion contraception

Four respondents previously used the IUD as a post-abortion contraception after repeated abortions. A respondent exclaimed: *“They rather gave it to me because of the abortion and they didn’t sell it to me. It was free after I did the abortion to protect me” (39 year old widow, on Copper-T 380A for 4 years).* The other respondents however paid for the IUD separately.

### Problems or side-effects experienced with using IUD

Generally, findings showed that at least, each woman (study participant) suffered from one of several side effects of the IUD after a few weeks of insertion. For some, the side-effects was severe and sufficient to warrant a removal of the IUD. An illustrative summary of the reported side-effects and reasons for IUD removal is outlined in Table 4.

**Table 4:** Summary of side-effects of IUD and reasons for removal

Reported Side-effects with IUD experienced	Reported reasons for removal of IUD
• Heavy bleeding	• Heavy prolonged bleeding
• Foul vaginal discharge (sometimes coloured)	• Vaginal discharge
• Infections	• To give birth
• Abdominal cramps	• Abdominal cramps
• Spotting bleeding	• Spotting or irregular bleeding
• Prolonged bleeding	• Infections
• Irregular bleeding	• Feeling of device removing
	• Discomfort with strings
	• Partner disapproval of FP method

The results indicate that reasons for removal of the IUD do not differ substantially from the side-effects experienced by respondents. Other reasons that led respondents to remove the IUD involve partner disapproval with the use of a FP method. One of the respondents explained that her partner disapproves of FP and so she was scared that although she has the IUD, the partner might notice the IUD and there might be a problem with that. She states:

*“My fear is maybe what if you are making love with your husband one day and the IUD gets remove by itself. Sometimes it bothers my mind and I’m scared when having sex” (*31year old married woman on Copper-T 380A for 8 months).

The physical features of the IUD also deterred some participant from continued use of the method. A respondents indicated that:

*“I think the uneasiness of that thread thing makes me dislike it. You know, once you’re cleaning it wants to come out and you have to push it there, you know… it has to be in there, it doesn’t need to stick out so that brings uneasiness” (47year old married woman on Copper-T 380A for 2 years).*

Another respondent said:

*‘Why should I allow that nasty thing to be put in my body? The shape of it alone puts me off so I will always go for another decent method and certainly not the IUD’ (25 year old unmarried woman using the injectable for 3years)*

Three respondents removed the IUD to give birth, but two of them categorically expressed their intentions of inserting the IUD again after birth. They both indicated their intentions as follows:

*“I removed the IUD to have another baby, but right after the pregnancy, I will wear the IUD again to protect myself from unplanned pregnancy because it is good for me” (39year old widow, who previously used Copper –T 380A for 4years).*

The other respondents said:

*“---even this when I was coming to remove it, the nurse asked me [laughing] ‘why do you want to remove it’ and I said I want to give birth again to two children. I think my partner is okay with the IUD and I will have it again after delivery” (38year old married woman, who previously used copper –T 380A for 2 years).*

### Reasons for IUD use among ever IUD users

Many reasons were noted for the use of IUD among the respondents who have ever used an IUD. Key among the reasons for women’s use of the IUD however pertained to a desire for a long acting FP method compared to short term FP methods. Summary of reasons given are arranged in order of most frequently mentioned in (Table 5).

**Table 5:** Illustrative summary of reasons for IUD use among ever IUD users

No.	Reasons for IUD use among ever IUD users	Frequency	Percentages (%)
1	Desire for long acting family planning method	18	24
2	Avoid unpleasant side effects with other FP types	15	20
3	Avoid forgetfulness to take pill	12	16
4	No hormonal influence	9	12
5	Perception of no side effects	7	9
6	Protect against pregnancy	5	7
7	Provider influence	4	5
8	Preference for an ‘obscure’ FP (‘obscure = hidden’)	3	4
9	Health reasons	2	2
10	Prevent worry	1	1
Total		76	100

To satisfy a desire for long acting family planning method, some respondents went out at all lengths to ensure that they had their desired FP method of choice. A respondent explained that:

*“I first went to a public hospital in Kumasi which had only short term FP methods. I wasn’t really convinced so I heard about the IUD from a NGO facility which I visited just for something more permanent. So I just walked in she took me through all the methods and after my last baby I was advised on that as the best option based on being hypertensive” (47 year old married woman on Copper-T 380A for 8 years).*

Another respondent said:

*‘I actually brought the LNG-IUS from the USA to be inserted for me here in Ghana because I really love it but very expensive to be inserted in the USA’ ( 23 year old unmarried woman on LNG-IUS for 2 years)*

Other respondents also mentioned avoidance of side effects with other FP methods and reduced forgetfulness in taking the pill. One respondent reported:

*“Oh yeah I love the IUD because there was nothing like I have forgotten to take my pills, I have forgotten to do this or that. It was just convenient I didn’t have to worry about anything. I didn’t have to worry. Once it was there and I was assured that it was 99% safe. So I didn’t worry” (….38 year old married woman, on copper-T 380A for 2 years).*

Two accounts from two women which capture their decision to use the IUD is presented in the following:

*“The fact that it’s inside makes me satisfied. I don’t have to see it, apart from the little pain I will experience over the months, I’m done. I can’t be faithful to that, taking the pill” (….26year old single woman ever used Copper-T 380A)*

The other respondent indicated that:

*“Because I was told it has nothing to do with the hormones and cells in me I think it is normal” (32year old married woman, ever used Copper-T 380A).*

### Perception of health risks associated with using the IUD

Mixed responses were obtained when the question on perception of health risk was asked. One woman feared that the IUD could cause cancer. According to her:

*“When I was going I didn’t know what to do and you know I was asking a lot of questions so I was going on the internet and my elder sister who passed away last year was telling me “Don’t do it!. Don’t do these things, they give cancer. “She died out of cancer but she said “don’t do it again”. They give cancers, the one…that thing insert here two things affect your blood and you get bloated up. You know, there were lot of fears” (.47year old woman on LNG IUS for 3 years).*

Another woman believed that though the IUD was effective in preventing pregnancy but the risk of contracting infections through the IUD makes it not to be totally safe. She explained that:

*“When it comes to health risks, I don’t know how you want me to; .oh you will still get STIs, you will still have seamen coming into you. You will still get the STIs” (….30 year old woman on Copper-T 380A).*

Another risk as mentioned by a 26 year old woman pertained more to a misconception about the IUD moving around the body into other organs by itself after insertion. She also mentioned the probability of experiencing perforation in the uterus when IUD is inserted. Although a rear occurrence with trained and competent providers the probability of perforation has been reported in clinical incidents relating to IUD insertion due to provider errors and use of rigid instruments among others. In the respondents own words she indicated that:

*“With the health risk you know when you put it on, when you insert they have this nylon thread, that they wrap it, sometimes it comes off as it helps the IUD to move around. You may not know, sometimes there may be some perforation or something. It may hurt u. I think that is, that makes it the only problem but aside that, it’s not a problem”.*

However, this same woman maintains that “*I don’t think there are any health risks, if u have an IUD inserted u don’t let everybody play around that part because if their fingers are…., I think the health risk is more about us and how you take care of yourself”.*

One respondent experiencing vaginal discharges was concerned that the discharges were due to the Copper IUD she had used for the past 2 years and might be risky to her health. She said:

“*Yeah, as I was saying about the discharges, I fear it could lead to something else” (…32year old single woman on Copper-T 380A)*

According to another woman, she was of the view that women risk having an ectopic pregnancy after using the IUD as a FP method. She explained saying:

*“Obviously yeah. You risk ectopic. You risk ectopic pregnancy I know that. I may say it has been argued with the LNG-IUS because of the hormone they can cause issues of the hormone imbalance things like that. I didn’t experience it anyway” (40year old married woman, on LNG-IUS for 3 years)*

In contrast to some respondents’ stated fears about the IUD, two other women could not certainly tell whether using the IUD was risky to their health. One said:

*“health risk? Not as I know” (….38 year old woman on Copper-T380A).*

Similarly, two other women believed since they didn’t experience any side effects or problems with the IUD, and upon the recommendation of health providers, then there was no health risks associated with it. One of them indicated that:

“*I don’t know much but so far, no. I’ve been to two midwives and they themselves said they used that one. That’s what makes me go in for that one” (….31year old woman, o Copper-T 380A for 2 years).*

### Future intention to use IUD

Women were asked about their future intention to use the IUD, and from their responses, only five (5) declared their intention to use it again because it was good, useful and effective for them. One respondent said:

*“I will come for the same thing when God willing I give birth”…38year old married woman, 2 children*

Another woman also reported

*“Yes, but right after the pregnancy, I will wear the IUD again to protect myself because it is good for me”… 39year old widow, 4 children*

It was clear from the IDIs that some women wished the IUD would have worked for them because they preferred a longer acting method, and also since it was recommended by a provider. Unfortunately some respondents experienced heavy/painful menses and frequent expulsion when given the IUD hence had to have it removed two months after insertion. Two women reported:

*“It’s not all that safe since I had heavy and painful menses when I was on IUD. I mean I wish it had worked for me, honestly. I really wished it had worked for me, I mean I was really counting on it. The fact that I mean it will be there for the next 5 years, for the next 10 years, you are protected and all that, u don’t have a problem. I really hoped it worked”….(30year old unmarried woman ever used Copper-T 380A)*

The other said:

*“I was quite unfortunate to have had the IUD falling off by its self on three occasions so I had to discontinue its use. But if it was working for me, I would have loved it. Why? Because it doesn’t let me grow fat or anything. You know, you stay your normal and you know that after five years which is quite a long time, you can change it. Five or ten years I think for you to change it. If you feel some uneasiness, you just walk into the facility and you just take it off” (47year old woman, on Copper-T IUD for 4 years).*

### Women’s knowledge about the IUD

All family planning providers reported that women (also clients) have very little knowledge of the IUD pertaining to its mechanism of action, its physical features, how it feels, and the essence of the copper around it. They attributed women’s knowledge of the IUD from shared experiences of friends, and which is represented negatively. Thus, when they asked clients knowledge of the IUD, clients primarily stated their misconceptions, side-effects and myths neglecting the usefulness and efficacy. On provider said:

*“Sometimes, they can be influenced by their friends. If I’m giving it percentage wise, it’s about 30percent of them who know depo, IUD, secure and all those stuffs from their friends” (IUD provider)*

Another provider reported that:

*“When you ask them, they tell you yes, I’ve heard it, but I’ve not seen it so you let them touch it and feel how it is before you do it for them. They will ask you so what is it, the wire that we are seeing there, it is just plastic. So they hold it to see how flexible it is. This is just a copper that is wound around it and is nothing that is going to hurt them, or touch anything” (IUD provider).*

Two providers asserted that among women who had visited them for contraceptive counselling and uptake, less educated women tended to have no knowledge about the IUD compared to educated clients who sometimes search the internet to read and obtain knowledge of the IUD prior to their visit to the FP clinic. The availability of internet services on smart phones provides quick information to users in areas that have good reception. This enable clients seeking information on contraception to browse quickly for knowledge. The observation made however is, clients who don’t have such facilities regardless of their educational levels tend to rely on service providers and/or significant others for information on family planning services. On this same issue, three providers also mentioned that only few clients have heard of IUD, and out of this number, very few have ever seen or touched it before. A provider narrated her experience as follows:

*“Yes, have you heard about IUD? You ask. You came in here we do family planning, which one do you like? The person if she’s … She will tell you that, I don’t have any idea about family planning” (IUD provider).*

On the other hand, a male provider strongly professed that because clients have little knowledge of modern FP methods particularly, IUD, he mostly provided it for them based on their past medical history, parity and the number of previous deliveries. Thus, he directly influenced clients’ use of the IUD. He indicated that:

*“Well over there, if they come, most of them don’t know about the IUD. Theirs is that they are here to protect themselves against pregnancy or are here for FP. So I take them through all the methods and then I ask how many children they have. And most of them too they gave birth at my place so I already know their history. After the counselling they will ask me, out of the whole lot which one am I choosing for them? You see, they are such that they have that trust and belief in me, so anything I say they comply” ( IUD provider).*

### Women’s reasons for not using the IUD

From the interviews, providers stated that women’s reasons for not using the IUD is mainly because of the fear of inserting the IUD in the uterus, and fears about the IUD. Fears about the IUD pertain to myths and misconceptions, as well as fears that the IUD will affect their health in the long term.. A respondent narrated some misconceptions and fears as follows:

*“I learnt the IUD can walk to the heart. I also learnt a woman had a baby and the baby was carrying IUD in her hand. If you get IUD, you won’t have children again. They have been saying all these things. So I tell them, it is not true.”*

Other reasons such as male partners’ disapproval of IUD use, (and any other FP method); fear of male partners’ knowledge about the IUD, and the uncomfortable feeling of the strings during sexual intercourse. Poor knowledge base of the IUD, a strong mind-set and resistance to the IUD also deterred uptake.

Concerning fears about the procedure for inserting the IUD, a female provider explained that unlike the short term FP methods like injectables or pills which are unobtrusive, IUD is noticeable and can be felt by the partner through the strings which lie in the vagina during intercourse. There is therefore a greater chance of it been detected or felt by the partner even when the partner has no knowledge about it.

### Providers’ reasons for clients IUD removals

The main reasons for clients’ removal of the IUD from providers view is the side effects evident through bleeding, cramps, vaginal infection, and abnormal menstrual cycle. The second key reason is for reproduction. Other reasons providers cited are to prevent feuds with their partners in circumstances where the IUD was inserted without partners’ knowledge and approval; fear of IUD from moving to other parts of the body; fear of IUD causing fibroid and cancer after prolonged use and for other health reasons.

*“The reason for them taking it out, some of them they bleed even after. Some can bleed for the whole 28days. They become very anaemic, fatigued and weak. For some, it’s not all, but as soon as you take it off and put them on medication for a week, that thing ceases and you monitor that client for about three cycles, you see that the client will be happy; and have the normal menstrual flow. The second thing too is that, she will come with that problem, you will take it off and this will go and interfere with her normal menstrual cycle, then she will be having abnormal menstrual cycle; instead of 28 days, at times they can go 26, 40.” (IUD Provider)*

Another provider said:

*“Some of them they come in for it without the consent from their partners. So along the way, their partners find out. And it tends to bring a quarrel among them so they quickly come for it to be removed” ( IUD provider).*

A female provider categorically maintained that some women who came to remove the IUD did so for no obvious reason; neither were they experiencing complications except to change to a different FP method. She indicated that:

*“There are instances they don’t come with any complaint, they feel it has been there for quite a long time, so they feel they should take it off and change to another method to see the best. Somebody who has been on the IUD for 7 years will come and take it off. Why do you want to take it off, and she tells you, I want to change it. It has been there for long so I want to change it. Just take it off and do something else for me” (IUD Provider).*

It was also noted that other women also wanted to test their fertility or reproducibility after prolonged IUD use. According to an IUD provider’s account:

*“They think once is been there for long without them getting pregnant, they should remove it and see if they are still fertile. And so they will remove, they will get pregnant and then they will come for help” (IUD Provider).*

### Client- provider- relationship in health facilities during FP counselling

All FP providers unanimously agreed having a good positive relationship with clients during contraceptive counselling. They described the counselling sessions as cordial and open to allow clients to freely discuss their concerns, and ask questions about the IUD, as well as other FP methods. Counselling is done in an open manner in a language which clients understand to facilitate communication.

### Ways to encourage IUD uptake among women

Currently, education on family planning services is integrated into general sexual and reproductive health promotion and education programmes at the local, regional and national levels. The scope of the sexual education at the public level is basic information on human reproduction and mechanism of how contraceptives work. Although there is a national family planning standards and protocols guiding family planning educational activities, the implementation of this protocol is determined by who is doing the education, the target population, mode of education and the level of theoretical and practical experience of the educator. In this regard, comprehensive education on long acting and reversible contraceptives as well as permanent methods looking at medical eligibility, side effects and complications are usually reserved for clinical trainings of providers and not the general public. Consequently, the limited scope of IUD education compels potential clients’ new users or adopters of IUD to rely on self-education by reading or information from significant others some of whom have limited knowledge shrouded with some of the identified myths and misconceptions that prevents IUD use and encourages discontinuation when side effects occur among users.

To encourage IUD use among women, all the FP providers suggested a holistic education and public sensitization. The holistic education as reported encompasses information on eligibility, side effects and complications for women on the IUD, as well as other long term FP methods for informed decisions on choice. Comprehensive education in this regard, should be carried out through the media (print and electronic) and mobile information vans particularly in rural communities. Additionally, messages should be targeted at dispelling the myths and misconceptions about the IUD. Sexually active young individuals should also be educated at the junior high school level on IUDs for future decision making. FP providers also recommended the use of text messages to promote IUDs on social media as a medium for reaching out to young people most of whom appears to frequent social media in recent times.

The sole male provider interviewed in the study stressed that public education aimed at promoting IUD adoption should consider the social context within which services are provided to encourage positive community attitudes, especially in areas where there is strong resistance or opposition to contraceptive practices. This will involve working with key opinion leaders on values clarification and satisfied clients in these communities to share experiences with IUDs. A female provider working at one of Marie Stopes centre however lamented that measures put in place by the organization to increase awareness and knowledge of long acting FP methods was inadequate. This she further explained that the activities being implanted to increase awareness and knowledge of long acting FP methods are ‘above the line marketing strategies’ which do not necessarily translate into uptake of services since there are issues of behavioural change and modification that must equally be addressed to compliment the efforts being made in the area of education and awareness creation at the community level to ensure high uptake.

The need for effective counselling that focuses on the positive aspects of the IUD was recommended to encourage interest and uptake among women. According to an IUD provider she indicated that:

*“ for counselling to be effective, we need to tell them the benefits of IUD, it saves time, it saves your money, it makes you do your house chores. There’s always peace at home, family are happy. Economic situation, because now the economy, when you insert IUD, is for long term. It doesn’t prevent you from doing your normal duties. The time that you waste here to come and do the 3 months, sometimes you tend to forget. When you insert IUD, you are at peace. You get time to do your real job” (IUD provider).*

On the contrary a provider indicated the relevance of informing clients about side effects and possible complications of IUDs as well. The provider stated that:

*“some IUD providers don’t tell the clients everything about the method they only tell them the benefits without informing them about the side effects and complications. So when the clients leave and have any bad effect they lose trust in the method and provider and rush back to have it remove. They won’t remove it if well informed” ( IUD provider)*

## Discussion

This study aimed to explore determinants of IUD use among women currently using the IUD, and women who had ever used the IUD; explore women’s experiences with the IUD pertaining to perceptions, side effects; reasons for removal; health risks, and future intention to use the IUD. Also, another objective was to explore level of knowledge of IUD among all women using a modern family planning (FP) method.

Findings show that women’s reactions and perceptions of the IUD was shaped by and associated with prior knowledge on the device, myths, fears and misinformation that they had heard about the IUD from their friends despite their full awareness of the importance of contraceptive use. Two-thirds of IUD users and few past IUD users were scared to consider the IUD as a FP method upon initially hearing of it. To a large extent, inadequate knowledge of the IUD, reinforced by general myths surrounding use of modern FP methods accounts for low uptake of the IUD. Some studies also show that misinformation, and lack of correct knowledge results in low uptake of LARC [22]. Women’s perceptions and knowledge are therefore shaped by these myths which further discourage contraceptive use and lead to open and incessant negative expressions of contraception.

Women’s negative perceptions about the IUD corroborate with providers responses. Providers stated that the fear of how IUD was inserted, misconceptions and fears about the IUD based on myths deterred women from accepting the IUD as a FP method. Yet, women who wanted to space, limit or stop childbearing had positive perceptions of the IUD. Similarly, women who preferred the IUD as FP method after being encouraged by providers held positive attitudes about it, in contrast to those who expressed side effects with it. Proper and effective counselling focusing on the benefits/ advantages of the IUD should be provided to women desiring to use a long acting reversible, and possibly, non-hormonal contraception to prevent pregnancies.

Lack of adequate knowledge may prevent IUD use; however, when women believe that FP providers are knowledgeable and can be trusted to maintain confidentiality, advice on method use, side effects and potential health risks. Women will be more receptive and convinced to take up the IUD as a first choice FP method. FP providers therefore have a major role to play in encouraging positive attitudes towards using the IUD through counselling.

On the contrary, some findings from the study show that because some clients are ignorant about family planning methods, there are elements of provider biases during counselling which leads to some providers either advising clients to use IUD based on their personal experiences or preference for IUD. In some reported instances the providers ‘forced’ clients to have an IUD as a post abortion contraception. This observation defeats the purpose of family planning counselling as it does not encourage choice and medical eligibility for IUD. Unannounced continuous supportive supervision of family planning service providers in their facilities will provide a valuable opportunity to identify gaps and provider biases during family planning counselling for further provider refresher education and updates on family planning counselling and decision making.

Women’s reasons for using the IUD varied slightly among current and ever users. Among current IUD users, preventing unwanted pregnancy emerged as the most important motivating factor, whilst the desire for a long acting FP was mentioned most often among ever IUD users. Although the ever IUD users would have loved to continue using the IUD, unbearable side effects of heavy menses and cramps lead to discontinuation. A further probe on this assertion shows that the type of IUD being referred to is the Cupper T 380^A^. There were few reported instance were clients opted for LNG-IUS but was not available in the facility. Perhaps, the availability of LNG-IUS (hormonal IUD) in facilities would have been preferred over Copper-T 380A which might have increased IUD usage in the study area. This suggests that among ever IUD users, there is an unmet need for LNG-IUS (hormonal IUD) which calls for programming and contraceptive security. The observation that IUDs have shown no or minimal reported side effects is an opportunity to use such satisfied clients for public education on IUDs to ensure continuity and increasing patronage since clients will really choose an IUD due to their understanding on the duration and perceived benefits over other methods.

Two-thirds of women expressed desire for a long acting method of protection for convenience purposes, ease of use, and avoid contraception failure. Post abortion women currently using the IUD, including women who removed it to give birth expressed immense usefulness and satisfaction since using it. They considered it to be very effective in serving the purpose they desired without side effects. There is evidence demonstrating the efficacy, safety, and cost-effectiveness of the IUD in pregnancy prevention [23, 24]. Other determinants of IUD use include: to prevent abortions; preference for an ‘obscure’ FP method; non-hormonal benefits; provider influence and encouragement; health reasons; protection from contraceptive failure; freedom from emotional worries; ease of use in order to focus on work and school.

The side effects of using IUD were reported as heavy prolonged bleeding, vaginal discharges, infections, spotting or irregular bleeding, and abdominal cramps. These are consistent with empirical evidence that IUDS are associated with irregular bleeding in some women [25].

Participants reported that continuous experience of these side effects for more than four months led to removal of the IUD. Findings are supported by providers who also attributed women’s removal of the IUD to mainly side effects. Women’s continuous experiences of side effects served as a barrier to future intention to IUD use. Among these women, some wished that the IUD worked for them, but felt that the side effects were indications of negative reactions, and the bodily system’s rejection of the IUD, as well as potential health risks with continued use.

Although none of the women suffering infections from using the IUD associated it with lack of personal hygiene, cross infection from partner infidelity, or poor insanitary lavatory conditions as common potential triggers of infections, there are other known causes of vaginal infections following IUD insertion among women which is important for providers to hint clients so that post IUD infections are not unnecessarily associated with these or the method. Similarly, understanding pre-exposure history of women regarding infections could enhance pre and post-method counselling.

Partners’ disapproval of FP use also contributed to removal of the IUD. Although this revelation is not new in our context through anecdotal evidence, it should be useful and practical to informing providers to find ways to encourage and educate male partners’ in contraception uptake. Few women who had ever used the IUD pointed out that, due to fear of negatively impacting their marriages decided to remove the IUD clandestinely. Some however noted that their partners were not informed about it, hence their decision to have it removed. In their view, the ‘strings’ heightened their dislike for the IUD, and to which their partners might notice.

Mixed views on perceptions of health risks associated with using the IUD were reported. Study participants thought having the IUD inserted might cause cancer, ectopic pregnancy, STIs, and uterine perforation. Although two-thirds of women thought otherwise, it is important that women are given appropriate and accurate information on all health complications that accompany each modern FP use. They should also be made to understand that individual differences, hormonal balance, and specific criteria pertain to the uptake of one modern FP method over the other.

The women in the study (a sample predisposed to knowing about FP by having visited a FP clinic before) knew of at least one modern method. This finding is consistent with previous findings in Ghana’s Demographic and Health Surveys (2008, 2014) which report universal contraceptive knowledge among all women. At least, all women mentioned three contraception types with the injectables and implants most frequently mentioned, followed by pills, condoms, and IUD. Less than one-third of the women interviewed mentioned male and female sterilization, and diaphragm. One study respondent mentioned the IUS as her current contraceptive method. Nine (9) out of the total selected women were currently not using any contraception. Social and cultural norms governing fertility, in addition to fear of contraceptive side effects, myths, and health risks may hinder contraceptive use. Demand creation through mass education with FP providers needs to be intensified and promoted among women in reproductive ages. Interventions should also focus on couples counselling to increase positive attitudes towards contraception for both partners.

It is also important to mention that from non-IUD users’ socio-demographic data, there is a high propensity for these women to increase their fertility since they are within active reproductive ages (20–35) but are not using any contraceptives due to unpleasant past experiences with IUD use. Although they all reported fears with using IUD again or any contraceptive, it is important that FP providers provide effective counselling for this group of women on other available and accessible alternatives of contraceptives to meet their FP needs.

Past IUD users who have switched from IUD to other FP methods are also highly susceptible to becoming pregnant should their contraceptive use become inconsistent/ irregular, or in the case of method failure. Only one woman out of the previous IUD users is currently using a LARC. Amongst the remaining, one-third is trying to become pregnant, while the rest are using a mix of short term and traditional methods due to the unavailability of preferred FP choice of implants (implanon NXT). In sum, the contraceptive history patterns of past IUD users are risky and raise concerns over unmet need for implants (implanon NXT) in the FP facilities.

## Implications

Results from this study has several programmatic and policy implications for improving uptake of LARC, especially IUD for women in all reproductive ages. Programs should:

- Focus on using social media to debunk myths and misconceptions that people have about LARC, particularly, IUD. Use of social media should be sensitive about the mode of communication. It should be in a language that people can understand and relate to, whiles considering the social context and social exchange systems.
- Design interventions (for instance, community interventions) to promote uptake of LARC methods and enhance existing service provision channels to provide accurate information and services on IUD in order to make it accessible, and available to young women desirous of LARC. Community interventions should be sensitive to the social context and appropriate medium of communication.
- Sensitize all persons visiting MSI centres on FP methods and post-abortion contraception through distribution of brochures and FP documentaries on video tapes. Documentaries should be comprehensive, and focus on advantages of IUD use, safety, efficacy and side effects to increase and sustain positive attitudes.
- Ensure the provision of comprehensive sexuality education for young girls and boys at senior high schools to equip them with appropriate information on family planning, contraception, and birth control methods as means of regulating fertility, spacing and reducing births. The resent positive outcome of the advocacy drive on sexuality education in schools resulting in the inclusion of sexuality education in schools curriculum in Ghana is timely as this provides a good entry point for this policy implementation in schools for the desired results

A key policy implication is task sharing IUD insertion and removal with mid-level providers in Ghana to ensure greater provider availability in all health facilities to improve uptake. This is a fall out from table1 indicating few numbers of FP providers in the study facilities. The researchers believed there would have been higher provider numbers if an IUD task sharing policy is in place and being implemented.

## Conclusion

A number of factors influence the use or discontinuation of IUD in Ghana. Provider capacity building for in-depth client counselling is required to make informed decisions at the facility level. Various targeted messages and use of satisfied clients are also needed to dispel IUD related myths and misconceptions at the community level. A future large scale study is also required to investigate if there are any seasonal, socio-economic and demographic variations in FP uptake within health facilities in Ghana. This when done will provide further information to informed national FP programme and policy decisions.

## Abbreviations

ERC: Ethical review committee
FP: Family planning
GDHS: Ghana demographic and health survey
GHS: Ghana health service
IDI: In-depth interview
IUD: Intra uterine device
IUS: Intra uterine system
LARC: Long acting and reversible contraceptive
LNG: Levongesterol
